# Prevalence and Correlates of Complementary and Alternative Medicine Use among Patients with Lung Cancer: A Cross-Sectional Study in Beirut, Lebanon

**DOI:** 10.1155/2017/8434697

**Published:** 2017-08-24

**Authors:** Farah Naja, Bilal Anouti, Hibeh Shatila, Reem Akel, Yolla Haibe, Arafat Tfayli

**Affiliations:** ^1^Department of Nutrition and Food Sciences, American University of Beirut, Riad El Solh, Beirut 1107 2020, Lebanon; ^2^Department of Internal Medicine, Division of Hematology/Oncology, American University of Beirut Medical Center, Riad El Solh, Beirut 1107 2020, Lebanon

## Abstract

Patients with lung cancer are increasingly seeking complementary and alternative medicine (CAM) to improve their physiological and psychological well-being. This study aimed to assess CAM use among lung cancer patients in Lebanon. Using a cross-sectional design, 150 lung cancer patients attending the Basile Cancer Institute at the American University of Beirut Medical Center were interviewed. Participants completed a questionnaire addressing sociodemographic characteristics, lung cancer condition, and use of CAM. The main outcome of interest was “use of any CAM therapy since diagnosis.” Prevalence of CAM use was 41%. The most commonly used CAM modality among study participants was “dietary supplements/special foods.” Results of the multiple logistic regression analyses showed that CAM use was positively associated with Lebanese nationality and paying for treatment out of pocket and was negatively associated with unemployment and having other chronic diseases. About 10% of patients used CAM on an alternative base, 58% did not disclose CAM use to their physician, and only 2% cited health professionals as influencing their choice of CAM. This study revealed a prevalent CAM use among lung cancer patients in Lebanon, with a marginal role for physicians in guiding this use. Promoting an open-communication and a patient-centered approach regarding CAM use is warranted.

## 1. Background

Lung cancer is the most common cancer in the world, representing 12.7% of all new cancer cases [1]. The majority of these cases occur in less developed countries (55%) [2]. Although lung cancer incidence rates and mortalities are still low in many countries of the Middle East and North Africa region (MENA) as compared to Europe or United States of American (USA), recent estimates are showing that these rates are gradually increasing in this region [3]. For instance, in Lebanon, among males, the prevalence of lung cancer was second to bladder cancer in 1986; however more recent data (in years 1992 and 2004) showed that lung cancer became the most prevalent malignancy [4, 5] with age-standardized incidence rates reaching 31.8 per 100,000 in 2008 [6]. In addition to its high prevalence in the world, lung cancer ranked first as the most common cause of cancer death, estimated to be responsible for one in five deaths (19.4% of the total) [7]. Such a high mortality persisted despite the recent significant advances in medical treatment and management [8–10]. Possible explanations could be due to the fact that conventional treatments for lung cancer, including radiation, chemotherapy, and surgery, are all associated with significant side effects such as fatigue, cough, pain, difficulty breathing/breathlessness, loss of appetite, trouble sleeping, weight loss, nausea, difficulty concentrating, anxiety, and depression [11]. Individuals living with lung cancer experience a disproportionate higher number of symptoms compared with other types of cancer, possibly because their disease is more advanced at diagnosis [12].

The side effects associated with conventional treatments of lung cancer are of major concern for the patient and may lead to decreased compliance and adherence to treatment [13]. In addition, many patients in developing countries are diagnosed at an advanced stage of the disease where conventional therapy may be less efficient [14]. For these reasons, patients with lung cancer are increasingly seeking alternative forms of treatment [13, 15].

Complementary and alternative medicine (CAM) is defined as “diverse medical and health care systems, practices, and products that are not generally considered part of conventional medicine” [16]. It is classified in four main groups: mind-body medicine which increases the mind's ability to affect bodily function and symptoms (e.g., guided imagery), biologically based practices using substances found in nature (e.g., herbs), manipulative and body-based practices involving manipulation or movement of body parts (e.g., massage), and energy medicine involving use of energy fields (e.g., Reiki) [16].

CAM use is popular among all patient populations [17]. According to the WHO, over three-quarters of the globe's population has been reported to use a form of CAM for the purpose of treatment of chronic conditions [17]. A few studies suggested that cancer patients use more CAM than the general population [18, 19]. In the USA, up to 90% of cancer patients used a form of CAM [20–23]. A cross-sectional survey of patients attending outpatient cancer clinics in Palestine reported a 69.5% prevalence of CAM use [24]. In Australia, recent studies reported that 30%–65% of cancer patients used CAM [25, 26]. A similar rate was reported in Jordan, where a survey of cancer patients showed that the prevalence of CAM use was 35.5% [27]. Concerning lung cancer patients, few studies have explored their use of CAM and the prevalence rates reported in the literature ranged between 4.3% in Greece [28] and 54% in Germany [29]. The popular use of CAM in cancer patients presumably reflects the benefits, real or perceived, by those who use them [30]. Patients are either “pushed” towards CAM because of dissatisfaction with conventional medical treatment or “pulled” towards it because of their philosophical beliefs and values [31]. Specific reasons suggested to explain such a common use of CAM included patients wanting to take responsibility for their own health, to relieve symptoms, to promote health and increase healing [32], to increase hope and personal control, to seek closer provider relationships, and to improve physiologic and psychosocial well-being [33].

The popular use of CAM among cancer patients raises concerns regarding its safety and potential interaction with conventional treatment. While certain CAM modalities were found to have beneficial effects, others had deleterious consequences for health and well-being of patients. For instance, acupuncture and acupressure were shown to relieve chemotherapy-induced nausea and vomiting [34, 35]. In addition, acupuncture, hypnosis, therapeutic touch, and massage all led to a reduction in cancer-related pain [36, 37]. CAM use was also shown to contribute to an improvement in mental health and emotional well-being of cancer patients. For instance, yoga, meditation, and exercise were found to reduce stress in cancer patients [18, 38]. On the other hand, the use of some forms of CAM among cancer patients could lead to unanticipated harm. Some CAM practices may worsen the side effects of conventional therapies and thus may negatively influence the compliance to conventional treatment [39].

One of the fastest growing markets of CAM products in the world has been documented to be within the MENA region [40]. In Lebanon, a small country of the MENA, this market is largely unregulated and could be subject to abuse by both patient and provider of CAM [41]. Given the high prevalence, burden, and mortality of lung cancer in Lebanon, obtaining information on the prevalence and correlates of CAM use among this patient population is important to influence physicians' priorities in advising their patients regarding the benefits and side effect of CAM use and help prioritize research investigating the efficacy and safety of CAM use. Therefore, the main aim of this study was to assess the prevalence and correlates of CAM use among patients diagnosed with lung cancer in Beirut, Lebanon. The specific objectives of the study were to assess the prevalence, characteristics, and determinants (demographic, sociocultural, economic, and medical) of CAM use among lung cancer patients as well as to examine the role of the physician in influencing CAM use in this patients' population.

## 2. Methods

### 2.1. Study Design and Participants

This was a cross-sectional survey assessing the prevalence and correlates of use of CAM among lung cancer patients attending the Naef K. Basile Cancer Institute at the American University of Beirut Medical Center [AUBMC]. Naef K. Basile Cancer Institute at AUBMC is the largest cancer center in the country with 2,500 new cancer patients evaluated each year, 13,000 hematology/oncology clinic visits, 14,000 patients treated at the ambulatory treatment unit, and 800 patients treated with radiation therapy. For this study, patients followed up in clinics at Naef K. Basile Cancer Institute were invited to participate. Inclusion criteria were patients older than 18 years of age, conversant in either the English or Arabic language, and diagnosed with lung cancer for at least 2 months. The 2-month duration would allow enough time for patients to explore the different CAM modalities. Patients were excluded if they were unable or unwilling to give consent for participation in the study. The study protocol has received ethical approval from the AUB IRB, under the protocol name IM.AT1.21. Sample size calculations showed that a total of 151 subjects were required in order to allow for an estimation of a 50% prevalence of CAM use at 95% confidence interval with an error margin not greater than ±8%.

### 2.2. Subject's Recruitment Protocol

Recruitment of patients took place at the clinics of Naef K. Basile Cancer Institute at AUBMC. Subjects meeting the inclusion criteria were briefed by the physician about the study. Subsequently, only patients who expressed willingness to participate were approached by the trained research fellow and were invited into an allocated private room in the clinic, where the consenting process and interview took place. IRB-approved consent forms were provided in English or Arabic based on the patient's preference. All of the research fellows/interviewers involved in this study had successfully completed the Collaborative Institutional Training Initiative (CITI) course as per the requirements of the Institutional Review Board (IRB). The research fellows assured the patients that confidentiality was highly respected and any information collected will not be shared with their health care providers.

### 2.3. Survey Instrument

In a face-to-face interview with the research fellow, participants completed a multicomponent questionnaire. The questionnaire was comprised of three sections: the first section includes questions assessing sociodemographic characteristics of the study participants such as age, gender, nationality, marital status, educational level, employment status, type of health insurance, and household income. The second section included questions specific to their lung cancer diagnosis, such as the duration of cancer, current state of the cancer, family history of lung cancer, and presence of other chronic diseases such as cardiovascular diseases (CVD) or chronic obstructive pulmonary diseases (COPD). The last section of the questionnaire included questions assessing the type of CAM treatments used, their mode of use (as complementary or alternative to conventional medical treatment and their frequency of use), their cost, perceived side effects, reasons of use, disclosure of CAM use to the physician, his/her reaction to its use, and what the main factors influencing the choice of CAM were. The content validity of this questionnaire was confirmed by a panel of experts consisting of one physician, one nutrition epidemiologist, and one health policy expert. The questionnaire was originally written in English, was translated to the Arabic language, and then back translated to English. The original and back-translated English versions of the questionnaire were examined to ensure parallel form reliability.

### 2.4. Data Analysis

The main outcome of this study was to determine the prevalence of CAM use among lung cancer patients. Patients were classified as users or nonusers of CAM based on whether they have used any form of CAM since diagnosis or not.

Proportions were used to describe the correlates and characteristics of CAM use. The two groups (users and nonusers of CAM) were compared using a Chi-square analysis. The associations of various sociodemographic and disease characteristics with CAM use were examined using simple logistic regression analyses. The dependent variable in these regression analyses was the use of CAM. In order to adjust for possible confounders and evaluate independent effects of each variable on the outcome (CAM use), a multiple regression model was built, in which all sociodemographic and disease characteristics were used as independent variables. Statistical significance was set at a *p* value < 0.05. Statistical Package for Social Sciences (SPSS) software version 20.0 for windows program was utilized to analyze the data.

## 3. Results

Data collection took place over the course of one year, between September 2015 and August 2016. Out of 156 lung cancer patients invited, 150 agreed to participate and completed the questionnaire (response rate: 96%). The prevalence of CAM use after diagnosis with lung cancer among the study participants was found to be 41%.

Table 1 displayed the various characteristics of the study population and their association with CAM use. The majority of participants were males (71%), married (89%), and had some form of health insurance (72%). Out of each four patients, three were Lebanese. Other nationalities reported were Iraqi (20%) and Syrian (7%). Only 35% of participants were employed at the time of the interview. As for the characteristics of the cancer, 38% of patients reported being diagnosed with the disease for more than one year and 24% between 2 and 3 months. Almost half of the participants had a metastatic cancer (52%) and 24% had a family history of lung cancer. Of study participants, 45% suffered from additional diseases such as hypertension, CVD, or COPD (Table 1). Using Chi-square test, “age,” “employment status,” and “suffering from other diseases” were shown to be significantly associated with CAM use (Table 1). Specifically, a higher percentage of CAM users was noted among patients younger than 55 years as compared to those who are older (60% versus 37%). The highest proportion of CAM users was found among employed patients (52%), as compared to retired (40%) and unemployed (33%). The prevalence of CAM use was higher among patients with lung cancer compared to that among patients suffering from additional diseases (53% versus 27%).

These findings were confirmed by the results of the simple logistic regression, displayed in Table 2, whereby the factors significantly associated with higher prevalence of CAM use were an older age, employment, and not suffering from other diseases. More specifically, the results of this study revealed lower odds of CAM use in association with age > 55 years (OR: 0.39, 95% CI 0.17–0.87), being unemployed (OR: 0.44, 95% CI 0.19–0.98), and suffering from other diseases (OR: 0.34, 95% CI 0.17–0.68). The multiple logistic regression model used to examine the correlates of CAM use after adjustment showed that CAM use increased significantly among Lebanese patients compared to non-Lebanese (OR: 7.9, 95% CI 1.13–55.45) and among subjects paying out of pocket for the treatment of lung cancer (OR: 13.54, 95% CI 2.23–82.23). Similar to the findings of the simple regression, the multiple regression analysis also showed that CAM use was associated with employment and not suffering from other diseases [unemployed (OR: 0.19, 95% CI 0.034–0.89); suffering from other diseases (OR: 0.13, 95% CI 0.03–0.52)]. The multiple regression model had a Chi-square value of 28.80 with a *p* value of 0.01.

**Figure 1:**
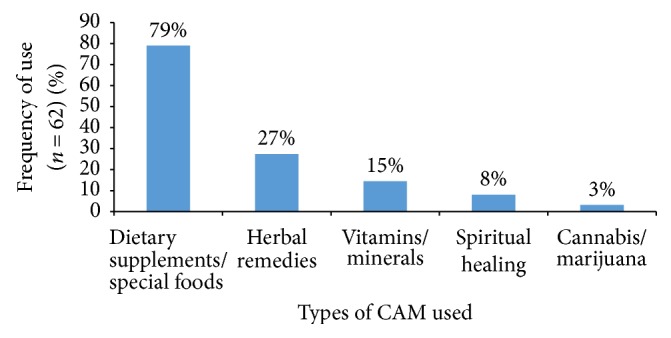
Types of CAM used in the study population (*n* = 62).

The different types of CAM used among the study population were illustrated in Figure 1. The most commonly used CAM was “dietary supplements (special foods),” followed by “herbal remedies,” “vitamin/mineral supplements,” “spiritual healing,” and “cannabis/marijuana.” The “dietary supplements (special foods)” category included graviola seeds, helia mushroom and milk, quinoa seeds and avocado mix, and cherimoya. “Herbal remedies” consisted of different kind of herbs such as Shita (herb found mainly in Morocco), thyme, lou'loub, spices, A'landa (from Palestine), and herbal teas. “Spiritual healing” category included various forms of prayers, visiting sanctuaries, seeking the blessing of various religious figures, and drinking “Zamzam water.” The latter is a type of holy water Muslims bring from Mecca.

Table 3 described the characteristics of CAM use among study participants. Almost 10% of patients indicated using CAM as alternative to conventional treatments and more than half reported not asking their doctor about use of CAM (58%). Among patients who asked the doctor, 46% said that the doctors' reaction to CAM use was encouraging while 19% said it was not. The two most common reasons reported for not disclosing CAM use to treating physicians were “not important, does not affect health” (47%) followed by “though it's important, I did not have the chance/time” (32%). When asked about the main influence of CAM choice, participants indicated friends (48%), media (40%), and personal choice (19%). Health practitioners were among the least to influence patients' choice of CAM (2%). The majority of CAM users (92%) reported experiencing no side effects associated with their use of CAM and 78% indicated that they would use CAM again.

When non-CAM users were asked if they would consider using CAM in the future, 70% said they would not. The main reasons for not using CAM were “lack of belief in the benefits of CAM” (61%) and the fact that the doctor did not prescribe it (33%).

## 4. Discussion

This cross-sectional study investigated the use and perception of CAM among lung cancer patients living in Beirut, Lebanon. The prevalence of CAM use among patients with lung cancer found in this study (41%) was comparable to estimates by previous reports. For instance, in a study examining CAM use among patients with lung cancer in European countries, prevalence rates were found to be 33.3% in Turkey, 37.2% in Spain, 40% in Denmark, and 42.9% in Israel [28]. In addition, in a study assessing CAM use among women with lung cancer in different institutions in the USA, 45% of patients reported using CAM [42]. However, lower prevalence rate was also reported in the literature, such as 25% in Sweden, 16.7% in Switzerland, 12.5% in UK, and as low as 4.3% in Greece [28]. On the contrary, relatively higher rates of CAM use were reported among lung cancer patients in Pennsylvania, USA, and Germany (50.9% and 54%, resp.) [29, 43]. Possible reasons for the variation in the prevalence of CAM use observed among different countries and geographical locations may be explained partly by differences in sociocultural perception of CAM use and disparities in the availability and access to conventional medicine. Moreover, differences in study design and definitions of what could be constituting CAM might have also contributed to the variation observed in prevalence of CAM use among lung cancer patients across countries [44]. It is important to note that the prevalence of CAM use among lung cancer patients found in this study was similar to that previously observed among a sample of breast cancer patients in Lebanon (40%) [45]. These prevalence rates (among lung and breast cancer patients) were higher than the prevalence of CAM use in the general population in Lebanon, estimated at the national level (29.87%) [46]. Such findings supported results of previous studies indicating that patients with chronic diseases are more likely to resort to CAM use compared to the general population [47, 48].

Similar to previous reports, findings of this study suggested that unemployment was associated with lower odds of CAM use [45, 49]. In addition, in this study, patients paying out of pocket for the cancer treatment were more likely to use CAM, as compared to those relying on a form of health insurance. In the Lebanese context, these findings suggested that CAM use among lung cancer patients followed a socioeconomic gradient whereby patients with a higher socioeconomic status (employed and paying out of pocket for their treatment) were more likely to use CAM. Such an association between a higher socioeconomic status and CAM use could be a reflection of the fact that, in Lebanon, CAM products and therapies are paid by the patient and not covered by available medical insurance policies [41]. In fact, in this study, more than 40% of patients indicated spending over 50 USD on CAM per month. For these patients, considering the GDP per capita estimated in Lebanon (8050 USD), the amount spent on CAM constituted a significant proportion (7.5%) [50].

In this study, patients with a Lebanese nationality were found to be more likely to use CAM than non-Lebanese patients, who were mainly Iraqis and Syrians. It could be argued that since the use of CAM in this study was found to be mainly influenced by friends and media as well as family beliefs, patients who are not Lebanese and are staying in Lebanon might be possibly away from their families and friends and hence were less influenced to use CAM. Furthermore, most of non-Lebanese patients come to Lebanon in order to receive conventional medical care offered at Naef K. Basile Cancer Institute at AUBMC and therefore may be more likely to report lower odds of CAM use compared to their Lebanese counterparts.

In congruence with earlier reports, in this study, the most common forms of CAM used by lung cancer patients were found to be dietary supplements/special foods and herbal remedies and vitamin/minerals [28, 43–45]. The high prevalence of use of these types of herbal CAM reflected the Lebanese and Arab ancient heritage that used the rich flora and herbal diversity of the region to create remedies for treating and curing diseases [51, 52]. The prevalent use of these CAM modalities could also be due to the general perception that they are natural and will not be harmful or have any side effects [45]. Patients tend not to be aware that many of the herbal preparations may contain dilute scores of different chemicals, the effects of most of which on cancer have not been documented [53]. In fact, some herbs may cause problematic interactions with chemotherapeutic agents, sensitization of the skin to radiation therapy, dangerous blood pressure swings, and other unwanted interactions with anesthetics during surgery [54].

In this study, 10% of patients reported using CAM as an alternative treatment. While this proportion remains alarming, it is important to note that it may be in fact an underestimation of the actual percentage of patients who may use CAM on alternative basis given that data was collected in a hospital setting, where patients were coming to receive a form of conventional treatment and would have been reluctant to report using CAM as alternative treatment. From a patient care perspective, this could cause delays in the start of and adherence to conventional cancer treatments [55]. Therefore, despite the low prevalence of alternative use of CAM therapies observed in the study, the repercussions could be significant with potential negative consequences on patients, their families, and the health system at large (increased cost of treatment). Among the factors reported to affect the decision of patients to resort to CAM use and decline conventional treatment were dissatisfaction with conventional medical practices [56], poor doctor-patient communication, frustration with the contradictory and consistently evolving state of current medical knowledge [57, 58], the increasing cost of conventional medical care [59], the intellectual and spiritual appeal of holistic models of health and healing [60], and the need for a sense of control over own health and self-management of the cancer [61].

In line with findings of previous studies examining the use of CAM among cancer patients, this study showed that 58% of patients did not disclose CAM usage to their treating physicians [42, 62]. When asked about the main reasons for not disclosing, patients in this study reported that CAM use does not need to be discussed with the physician. Furthermore, even when the patients wanted to discuss CAM use with the physicians they did not seem to have the time. The marginal role of the physician suggested by the findings of this study was further underscored by the fact that the majority of patients using CAM chose their CAM based on input from friends (48%) and media (40%) and only 1% relied on health practitioners. This is in accordance with the results of previous studies examining CAM use among various patient populations in Lebanon, including breast cancer, Type 2 Diabetes Mellitus, and infertility as well as pediatric leukemia [63]. The study on CAM use among lung cancer patients in different European countries also showed similar findings, where the most prominent sources of information for CAM choice were outside the medical system and included friends (65.4%), followed by family (30.8%) and media (23.1%) [28].

The findings of this study regarding a marginal role of the physician in the patients' choice of CAM use raise concerns regarding the physician-patient communication. The latter is increasingly considered as integral and an important part of cancer treatment [64], as it may protect patients from possible harmful effect of certain CAM therapies and improve adherence to recommended conventional treatment [65]. Mutuality and shared decision making between physicians and cancer patients could address the need of patients to have an active role in the treatment decisions and processes and hence may adhere better to the recommended treatment. Previous research suggested that the use of a simple screening question may allow patients who wish to discuss CAM to have the opportunity to disclose information and seek advice from their treating physicians [66]. It could be argued that physicians may feel uncomfortable receiving questions about CAM from their patients given their limited knowledge about the subject. In fact, a study of knowledge about CAM among registered health care providers in Sweden showed that communication between patient and health care provider regarding CAM was rare and that over 90% of participants had minor or no knowledge about CAM [67]. Therefore, integration of CAM modules in undergraduate and graduate medical education may help alleviate physicians' discomfort related to answering questions about CAM and enhance patient-physician communication regarding CAM use [68].

The findings of this study ought to be considered in light of a few limitations. First, the selection of one medical center might have affected the generalizability of the findings. However, the Naef K. Basile Cancer Institute at AUBMC is considered the largest cancer treating center in Lebanon (as evidenced by the high patient load) and is a major referral center for the treatment of all cancers from Lebanon and the region. Second, it is possible to conceive that the prevalence estimate of CAM use obtained in this study may be an underestimation of the actual prevalence since patients' recruitment took place in a clinical setting and hence they may have been biased towards conventional medicine use. In addition, the interviewer-based approach in data collection could have resulted in a social desirability bias; however research fellows were extensively trained to have a nonjudgmental and neutral attitude and used standardized techniques, avoiding questions that could influence the subject's responses [69].

## 5. Conclusion

This is the first study in the MENA region and Lebanon to examine the use of CAM among lung cancer patients. Findings of this study highlighted a prevalent use of CAM among lung cancer patients in Lebanon, with dietary supplements/special foods and herbal remedies, and vitamin/minerals being the most commonly used CAM modality. While older patients and those belonging to a lower socioeconomic status were less likely to use CAM, patients paying out of pocket for treatment or holders of the Lebanese nationality were more likely to use some form of CAM. Furthermore, the results of this study suggested a marginal role of the treating physicians played in orienting CAM use since the majority of the patients were found to rely on friends and media for choice of CAM and a significant proportion of patients did not disclose their CAM use to their physicians. The findings of this study revealed the need for a concerted effort to improve patient-physician communication and heighten awareness of both practitioners and patients with lung cancer on the proper and safe use of CAM therapies in adjunction with conventional treatment modalities.

## Figures and Tables

**Table 1:** Sociodemographic and disease-related characteristics of study participants by CAM use (*n* = 150)^*∗*^.

Characteristics	Overall *n* = 150	CAM users *n* = 62	CAM nonusers *n* = 88	*p* value^*∗∗*^
Age (years)				
≤55 years	30 (20)	18 (60)	12 (40)	**0.02**
>55 years	120 (80)	44 (37)	76 (63)	
Gender				
Male	106 (71)	44 (42)	62 (59)	0.54
Female	44 (29)	18 (41)	26 (59)	
Nationality				
Non-Lebanese	37 (25)	13 (35)	24 (65)	0.25
Lebanese	113 (75)	49 (43)	64 (57)	
Marital status				
Single	16 (11)	4 (25)	12 (75)	0.13
Married/live in	134 (89)	58 (43)	79 (59)	
Educational level				0.22
High school level	89 (59)	34 (38)	55 (62)	
University level	61 (41)	28 (46)	33 (54)	
Employment status^*∗∗∗*^				
Employed	52 (35)	27 (52)	25 (48)	0.05
Retired	47 (32)	19 (40)	28 (60)	
Unemployed	49 (33)	16 (33)	33 (67)	
Type of health insurance				
Insured (public/private)	108 (72)	41 (38)	67 (62)	0.12
Self-paying	42 (28)	21 (34)	21 (24)	
Monthly household income^*∗∗∗*^				
<1000$	55 (41)	20 (36)	35 (64)	0.11
≥1000$	78 (59)	38 (49)	40 (51)	
Disease-related characteristics				
Duration of lung cancer				
2-3 months	36 (24)	16 (44)	20 (56)	0.56
4–12 months	57 (38)	24 (42)	33 (58)	
>1 year	57 (38)	22 (39)	35 (61)	
Current status of lung cancer^*∗∗∗*^				
Early/locally advanced	46 (48)	19 (41)	27 (59)	0.55
Metastatic	49 (52)	21 (43)	28 (57)	
Family history of lung cancer^*∗∗∗*^				
No	110 (76)	45 (41)	65 (59)	0.45
Yes	34 (24)	15 (2544)	19 (56)	
Suffer from other diseases (hypertension or CVD or COPD)^*∗∗∗*^				
No	80 (55)	42 (53)	38 (48)	<**0.01**
Yes	66 (45)	18 (27)	48 (73)	

^*∗*^Values in this table represent *n* (%); ^*∗∗*^*p* values were derived from Chi-square tests; ^*∗∗∗*^missing data (the totals of these variables do not add up to 150).

**Table 2:** Correlates of CAM use among study participants using simple and multiple logistic regression analyses (*n* = 150)^*∗*,*∗∗*^.

	Univariate logistic regression	Multiple logistic regression
Age (years)		
≤55 years	1	1
>55 Years	**0.39 (0.17**–**0.87)**	0.61 (0.13–2.79)
Gender		
Male	1	1
Female	1.02 (0.50–2.09)	2.07 (0.46–9.20)
Nationality		
Non-Lebanese	1	1
Lebanese	1.41 (0.65–3.05)	**7.9 (1.13**–**55.45)**
Marital status		
Single	1	1
Married/live in	2.13 (0.65–6.94)	6.20 (0.31–125.49)
Educational level		
High school level	1	1
University level	1.37 (0.71–2.66)	0.67 (0.17–2.67)
Employment status		
Employed	1	1
Retired	0.63 (0.28–1.37)	0.82 (0.21–3.24)
Unemployed	**0.44 (0.19**–**0.98)**	**0.19 (0.034**–**0.89)**
Type of health insurance		
Insured (public/private)	1	1
Self-paying	1.64 (0.80–3.35)	**13.54 (2.23**–**82.23)**
Monthly Income		
<1000	1	1
>1000	1.66 (0.82–3.37)	2.57 (0.66–10.02)
Duration of lung cancer (*n* = 76)		
2-3 months	1	1
4–12 months	0.91 (0.39–2.11)	1.20 (0.22–6.42)
>1 year	0.79 (0.33–1.83)	0.39 (0.08–1.74)
Current status of lung cancer		
Early/locally advanced	1	1
Metastatic	1.07 (0.47–2.41)	0.95 (0.28–3.20)
Family history of lung cancer		
No	1	1
Yes	1.14 (0.52–2.48)	0.35 (0.07–1.62)
Suffer from other diseases (hypertension or CVD or COPD)		
No	1	1
Yes	**0.34 (0.17**–**0.68)**	**0.13 (0.03**–**0.52)**

^*∗*^Values in this table represent odds ratios (OR) and their corresponding 95% confidence intervals (CI). ^*∗∗*^Significant OR and their corresponding 95% CI are bolded.

**Table 3:** Prevalence and characteristics of CAM use in the study population (*n* = 150).

Prevalence of CAM use	*n* (%)
Used CAM in the previous year	
No	107 (71)
Yes	43 (29)
Used CAM since diagnosis	
No	88 (59)
Yes	62 (41)
*CAM related characteristics among CAM users *(*n* = 62)	
Alternative or complementary to conventional treatment	
Complementary	56 (90)
Alternative	6 (10)
Asked doctor about CAM used	
No	36 (58)
Yes	26 (42)
Doctor's reaction to CAM use (*n* = 26)	
Neutral	9 (35)
Encouraging	12 (46)
Discouraging	5 (19)
Reasons for not disclosing CAM use to doctors (*n* = 19)	
Not important, does not affect health	9 (47)
Though important, I did not have the chance/time	6 (32)
Doctors does not believe in it	2 (11)
Doctors does not have the expertise	1 (5)
Doctor will be angry	1 (5)
CAM choice^*∗*^	
Friends	30 (48)
Media	25 (40)
Personal choice	12 (19)
Family beliefs	8 (13)
Health food shop	2 (3)
Alternative medicine therapist	2 (3)
Health practitioner	1 (2)
Religious beliefs	1 (2)
Frequency of CAM use	
Only one time	7 (11)
Once per month	3 (5)
Regular (at least twice a week for a minimum of one month)	46 (74)
Other	6 (10)
CAM provider	
Purchased from local store/pharmacy	44 (76)
Naturopath	8 (14)
Practitioner of traditional medicine	2 (3)
Homeopath	2 (3)
Massage therapist	1 (2)
Secret dealer (cannabis/marijuana)	1 (2)
Estimated cost of CAM use per month	
<10$	16 (29)
10–50$	16 (29)
>50$	23 (42)
Reasons for CAM use^*∗*^	
To improve general health and ensure long term survival	35 (57)
To manage cancer complications/progression	32 (52)
Belief in advantages of CAM	27 (44)
To help in relaxation and feeling better psychologically	25 (40)
More natural practice	25 (40)
To reduce side effects of conventional therapy	24 (39)
To feel more in control over health	20 (32)
Curiosity	10 (16)
To provide energy	9 (15)
Family tradition/culture	9 (15)
Religious	4 (7)
How do you assess the usefulness of CAM?	
Not at all	14 (23)
Some	9 (15)
A lot, very satisfied	17 (27)
Can't tell	22 (36)
Side effects from CAM (*n* = 60)	
No	55 (92)
Yes	2 (3)
Undecided	3 (5)
Would you use CAM again? (*n* = 60)	
No	9 (15)
Yes	47 (78)
Undecided	4 (8)
Would you recommend CAM use to other lung cancer patients?	
No	13 (21)
Yes	40 (65)
Undecided	9 (15)

^*∗*^Several answers apply.
